# Objective gait analysis using Kinect v2® for the prognosis of walking during the acute phase of rehabilitation after proximal femoral fracture surgery

**DOI:** 10.3389/fresc.2025.1527825

**Published:** 2025-06-12

**Authors:** Kohki Matsubara, Gen Kuroyanagi, Atsushi Imamura, Yuichiro Mizuno, Shintaro Tsukada, Ruido Ida, Haruhiko Tokuda, Hideki Murakami, Hideki Okamoto, Yoshino Ueki

**Affiliations:** ^1^ Department of Rehabilitation Medicine, Nagoya City University Graduate School of Medical Sciences, Nagoya, Japan; ^2^ Department of Rehabilitation Medicine, Northern Mie Medical Center Inabe, Inabe, Japan; ^3^ Department of Orthopedic Surgery, Nagoya City University Graduate School of Medical Sciences, Nagoya, Japan; ^4^ Department of Orthopedic Surgery, Chuno Kosei Hospital, Seki, Japan; ^5^ Department of Metabolic Research, Research Institute, National Center for Geriatrics and Gerontology, Obu, Japan; ^6^ Department of Clinical Laboratory, National Center for Geriatrics and Gerontology, Obu, Japan

**Keywords:** proximal femoral fracture, rehabilitation, walking prediction, gait analysis, Kinect v2®

## Abstract

**Objective:**

While factors affecting gait post-surgery for proximal femoral fracture (PFF) have been studied, the prognostic value of objective gait analysis during acute postoperative rehabilitation remains unclear. Microsoft Kinect for Windows v2® (Kinect) is a noninvasive, low-cost, and easy-to-implement posture estimation device. However, its application in analyzing gait for these patients is underreported. This study aims to analyze gait during acute postoperative rehabilitation using Kinect, identifying key gait evaluation metrics and prognostic indicators.

**Methods:**

This study included 17 patients who were admitted to our hospital with PFF and underwent surgery. Rehabilitation began the day after surgery, with weekly Kinect-recorded gait videos from postoperative weeks 1 to 4. Gait parameters were analyzed thereafter. Participants were divided into two groups: those unable to walk with a walker one week after surgery were classified as having a poor prognosis (*n* = 7), while those who could were classified as having a good prognosis (*n* = 10). Various gait parameters were then compared between the groups to examine factors predicting gait prognosis. Furthermore, the hip joint pain/activities of daily living (ADL) index Oxford Hip Score (OHS) and the dynamic balance indicator timed up and go test (TUG) were evaluated, and the correlation with each gait parameter was examined. Key gait parameters were analyzed in both groups after equivalent rehabilitation periods.

**Results:**

Increased gait velocity, decreased gait cycle time, increased swing phase, decreased stance phase, and increased maximum hip joint flexion angle were observed during the postoperative rehabilitation in both study groups. Compared with the good prognosis group, the poor gait prognosis group showed worse swing/stance phases of either healthy or affected limb, TUG, and OHS. The mean TUG value and OHS correlated with each gait parameter. Even after the same rehabilitation gait training period, the good prognosis group showed greater improvements in gait velocity, swing phase, stance phase, hip and knee joint flexion angles, and OHS compared to the poor prognosis group.

**Conclusion:**

During the acute phase of rehabilitation after PFF surgery, adequate hip pain control and early weight bearing of the operated limb are important for favorable gait prognosis.

## 1 Introduction

As the average life expectancy of the general population increases worldwide, the number of proximal femoral fracture (PFF) in older people is increasing (1). In Japan, more than 200,000 patients experience PFF annually and require surgeries (2). After PFF surgery, walking training is performed as part of rehabilitation. Numerous factors affect recovery of walking ability after PFF surgery, including walking ability before injury, age, sex, and degree of dementia (3). However, the prognostic factors based on objective gait analysis during acute postoperative rehabilitation are still unknown.

In human gait analysis, motion capture has traditionally been employed, using multiple cameras, markers attached to the body, and special suits. However, because this system necessitates a dedicated room and expensive equipment, it is only available in limited facilities (4). Microsoft Kinect for Windows v2® (Kinect) was developed in 2014 as a device that can control game consoles and computers using gestures and voice recognition. Posture estimation is performed from images obtained by an infrared emitter and a depth sensor, and three-dimensional (3D) skeletons can be detected and estimated noninvasively without markers by simply capturing a person on the camera (5). Kinect allows for the calculation of various gait parameters from the change in coordinates by simply capturing a person walking a few meters. In addition, the angles of each body joint can be easily measured from coordinate changes during walking without requiring complex 3D motion analysis (6). Many studies have reported that Kinect is a useful device for gait analysis in the medical field. Kinect-based gait evaluation for gait abnormalities and postural disorders highly correlated with clinical gait scales (7). Kinect v2® has been used to detect mild cognitive impairment and Alzheimer's disease by analyzing straight and curved walking (8, 9). It has also been reported that the Timed Up & Go Test, 10 Meter Walking Test, and real-time joint range of motion measurement can be measured with Kinect (10). Our previous research demonstrated that Kinect can evaluate gait parameters as accurately as conventional systems (11). However, based on our study literature, objective gait analysis using a single Kinect and the identification of prognostic factors during acute rehabilitation of patients with PFF have not been established.

In this study, we utilized Kinect to conduct a detailed gait analysis during acute rehabilitation following PFF surgery, focusing on evaluating gait and identifying key prognostic indicators. If objective gait analysis using Kinect can elucidate key factors for good gait prognosis and prevention of recurrent falls, the healthy life expectancy of elderly people will be extended.

## 2 Methods

### 2.1 Participants

The study participants were elderly people aged 60 years or older who visited our hospital for trauma between November 2022 and February 2024, were diagnosed with PFF, and were indicated for surgery with femoral head replacement or open reduction and internal fixation, and were able to walk independently or with a cane before trauma. Regarding the exclusion criteria, to investigate changes in gait due to the effects of PFF, we excluded patients with a medical history that could affect their walking ability after surgery. Specifically, patients were excluded if they were unable to walk before injury, had high-energy trauma, fractures in other body parts, head injury, history of conditions that affected their ambulation after surgery (e.g., cerebrovascular disease, neurodegenerative diseases, hip and knee joint diseases, psychiatric disorders, dementia, etc.), or had postoperative weight-bearing restrictions.

Recently, being able to walk with walking aid within the first week after PFF surgery has been reportedly important (12, 13). A retrospective analysis of the medical records of 228 patients with PFF reported that good walking ability one week after surgery was associated with a higher rate of direct discharge to home (12). Therefore, walking ability one week after surgery is considered important for good walking prognosis at the time of discharge. In this study, participants were classified according to their walking ability one week after surgery and an objective gait analysis was performed.

Rehabilitation training was started by the therapist on the first postoperative day. All participants were allowed to bear full weight. They underwent our standard rehabilitation training program for approximately 40 min per day. Depending on their pain level, participants were encouraged to get out of bed and received staged rehabilitation training including basic movements, range of motion training, wheelchair transfer, walking within parallel bars, walking with a walker or cane, and independent walking.

### 2.2 Instrumentation and gait analysis procedures

Kinect was used in this study. The participant's gait was captured by a Kinect v2 sensor, and color information at 1,920 × 1,080 (30 fps) and depth information at 512 × 424 (30 fps) were obtained. The data was transferred to a laptop (Inspiron 15-7568, Dell Inc.) and processed by the official software (Kinect for Windows Software Development Kit 2.0, SDK), and the 3D coordinates of each body part were recorded and generated skeletal model. The coordinates of Kinect of the spine shoulder, spine mid, spine base, hip, knee, ankle, and foot were estimated without markers.

**Figure 1:**
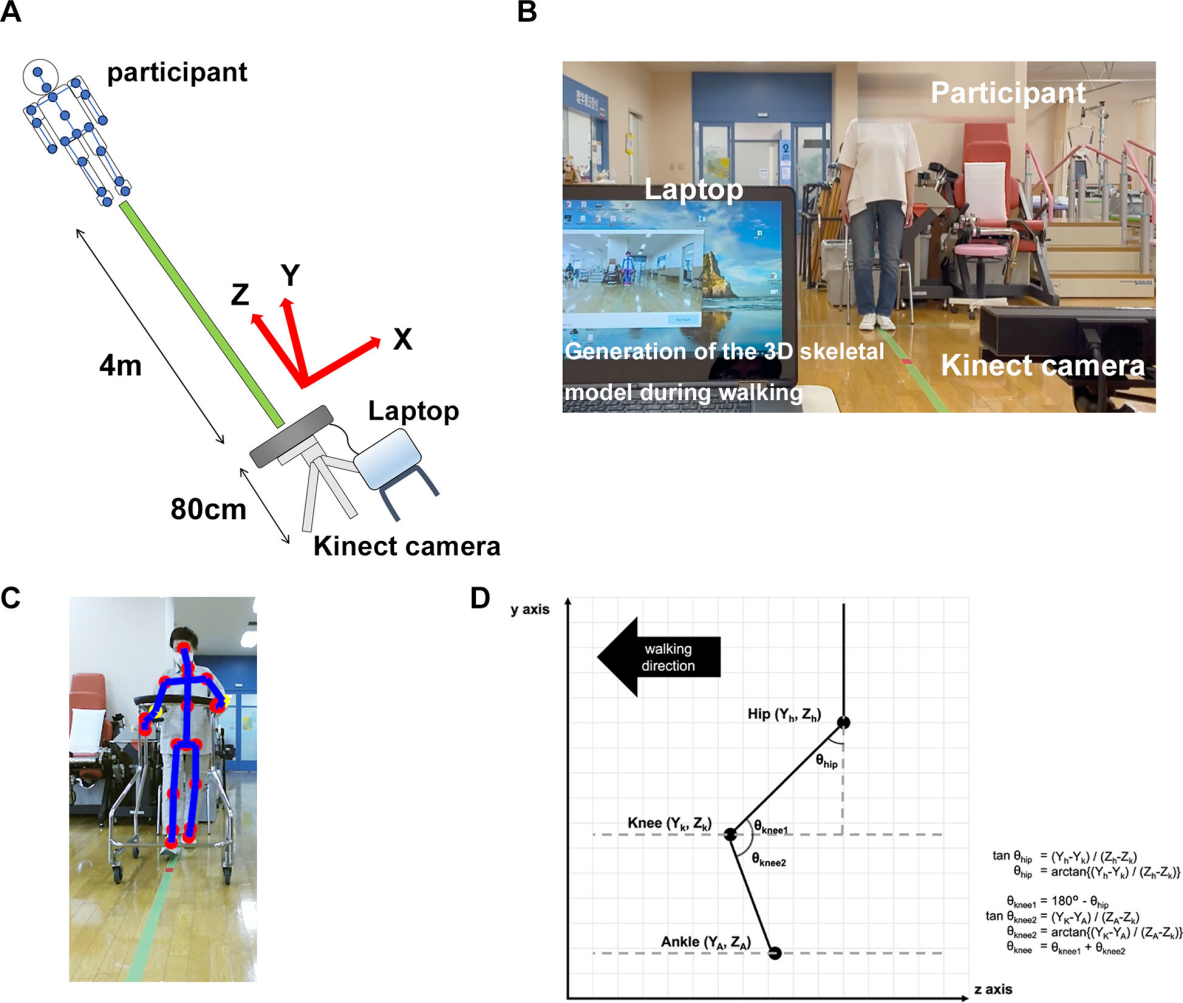
Methods of recording walking video and generating 3D skeletal model using Kinect. **(A)** Participants were instructed to free walk 4 m toward the Kinect camera with or without a walking aid (a walker or a cane). Kinect coordinate axes *X, Y*, and *Z* were defined as mediolateral, vertical, and posteroanterior axes, respectively. The *Z*-axis corresponded to the walking direction. **(B)** Representative image of the recording video during walking of the participants. Participants walked five consecutive round trips on one recording (total 40 m). **(C)** Representative image of the generated 3D skeletal model of the participants during walking with horseshoe type walker. **(D)** Calculation methods of the hip and the knee flexion angles from each coordinate.

Gait analysis procedures using Kinect were described previously (11). Regarding the installation of the Kinect, the tripod was installed in the same position in all sessions. Additionally, Kinect camera was calibrated to be 0 ± 1° relative to the floor using a spirit level. All participants walked a distance of 4 m from the walking starting point to the Kinect camera for 5 consecutive round trips (40 m in total) without taking a break on one recording. Gait analysis was performed from the video of the path from the starting point to the Kinect camera during the 5 consecutive walks. Participants ambulated unaided with or without a walking aid such as a walker or cane (Figures 1A,B). In this study, walking with a walker is essential for recording the walking video 1 week after surgery. The use of a horseshoe type walker allowed for video capture without obstacles, and enabled to generate 3D skeletal models of the participants target area from the spine to the foot during walking (Figure 1C). Walking video recordings using Kinect began 1 week after surgery and were taken once a week until postoperative week 4. The coordinates axis of Kinect was defined as *X, Y*, and *Z*, indicating mediolateral, vertical, and posteroanterior axes, respectively. The *Z*-axis corresponded to the walking direction. The velocities of each coordinate were calculated by dividing the amount of change (meter) in coordinates by 30 Hz (=1/30 s), and noise components of ≥6 Hz were removed by a low-pass filter for obtaining smooth waveform (11). Preprocessing was performed only with a low-pass filter in this study. From each 3D skeletal model coordinate, the joint angle was calculated using an inverse trigonometric function (Figure 1D). In this study, the hip flexion angle was calculated from the angle between *Y*-axis (vertical) and the hip–knee vector on the *Y*–*Z* plane. The knee joint flexion angle was calculated from the angle between the hip–knee vector and the knee-ankle vector on the *Y*–*Z* plane. The programming language Python 3 and its libraries numpy, pandas, and scipy were used for the conversion. The calculated gait parameters were as follows; gait velocity (m/s), gait cycle time (s), swing phase (%), stance phase (%), step length (m), stride (m), maximum hip joint flexion angle (degree), and maximum knee joint flexion angle (degree) (Detailed calculation methods were shown in Table 1). All gait parameters were calculated from the average of the extracted waveforms at five-time walking trials. In order to obtain parameters during steady walking, excluding the acceleration process, the first step from the start of walking was excluded. In addition, this was expected to have an effect that makes no difference depending on whether the participant starts walking from the healthy limb or the affected limb. Furthermore, except for the step length, all parameters were calculated separately for the healthy and affected limbs. The stance phase was defined as the period of ankle velocity <1 m/s on the *Z*-axis.

**Table 1:** Gait parameters and calculation methods.

Gait parameters	Calculation methods
Gait velocity (m/s)	Mean velocity of the spine base on the *Y*-*Z* plane
Gait cycle time (s)	The time between one ankle strike and the next on the *Y*-*Z* plane
Swing or Stance phase (%)	Percentage of the swing or stance phase in the total gait cycle [Definition] Swing phase: ankle speed to Z-direction is ≥1 m/s on the *Y–Z* plane Stance phase: <1 m/s
Step length (m)	The maximum distance between the left and right ankles during each gait cycle on the *Y-Z* plane
Stride length (m)	The distance between the ankle at the start of the gait cycle and the ankle at the end of the gait cycle on the *Y*-*Z* plane
Maximum hip joint flexion angle (degree)	The angle between the *Y*-axis and the hip-knee vector on the *Y*-*Z* plane
Maximum knee joint flexion angle (degree)	The angle between the hip-knee vector and the knee-ankle vector on the *Y*-*Z* plane

*X*, mediolateral axis; *Y*, vertical axis; and *Z*, posteroanterior axis.

### 2.3 Dynamic balance index and hip joint pain/ADL index evaluation

As dynamic balance indicators, the 3-m timed up and go test (TUG) and functional reach test (FRT) were measured once a week from postoperative weeks 2 and 4. These two tests could be used as a simple measure of balance comparable to the Berg balance scale (14). In addition, all participants were asked about OHS as a hip joint pain/ADL index once a week from postoperative weeks 2 and 4. OHS was developed as a specific evaluation scale for hip joint diseases and is mainly used for pre- and postoperative evaluations of artificial hip joints or artificial femoral head replacement (15, 16).

### 2.4 Statistical analysis

In a comparison between two groups, Student's *t*-tests were performed on each weekly gait parameter of the affected and healthy limb for all participants, and on each weekly gait parameter of the affected and healthy limb, dynamic balance index, OHS, mean age, and mean height for the good and poor prognosis groups. One-way analysis of variance (ANOVA) was used to compare differences in continuous data between three or more groups, followed by Shaffer *post-hoc* test. ANOVA was performed on each gait parameter, dynamic balance index, and OHS at 2, 3, and 4 weeks after surgery for all participants, and on each gait parameter, dynamic balance index, and OHS at 1, 2, 3, and 4 weeks or at 2, 3, and 4 weeks after surgery for the good and poor prognosis groups. In the parametric analysis, normality of the parameters was verified with Shapiro–Wilk test. Pearson's correlation analysis was performed between gait parameters in mean values of all participants and TUG or OHS. A correlation matrix was calculated using Pearson's correlation coefficient for the mean gait velocity of all participants and the mean values of each gait parameter (15 items in total), and a correlation heatmaps over time was created using GraphPad Prism for Windows (version 10.4.2). We investigated prognostic factors for walking based on an intergroup comparative analysis of all gait parameters in good and poor prognosis groups, which were divided according to whether the participant was able to walk with a walker without assistance 1 week after surgery. Power analysis was performed to examine the effect size for factors that showed significant differences in between-group comparisons. Fisher's exact test was performed for participant factors (Sex, systemic diseases, walking ability before injury, surgical methods) between good and poor prognosis groups. All statistical analyses were conducted using the R statics package, version 4.3.1 (R Core Team, Foundation for Statistical Computing, Vienna, Austria). The *P*-value <0.05 was regarded as significant.

## 3 Results

### 3.1 Information of all participants

In total, the study enrolled 17 participants, the average age was 78.5 ± 9.61 years, the male-to-female ratio was 5: 12, and the average height was 156.1 ± 6.66 cm. Ten participants comprised the good gait prognosis group, and 7 made up the poor prognosis group. Only age-related significant differences were observed between the two groups (Table 2). Age is one of the confounders that can affect gait performance. In this study, the poor prognosis group tended to be older than the good prognosis group. This may have also influenced recovery during postoperative rehabilitation. However, age was not considered as a confounding factor in the two-sample *t*-test performed in this study.

**Table 2:** Information of all participants.

Information of all participants			
Number	17		
Mean age	78.5 ± 9.61 years		
Sex	Male: 5 Female: 12		
Mean height	156.1 ± 6.66 cm		
	Good gait prognosis group *N* = 10	Poor gait prognosis group *N* = 7	Statistical analysis
Mean age	74.6 ± 10.21 years	84.1 ± 5.20 years	*P* = 0.039*, *r* = 0.504
Sex	Male: 4 Female: 6	Male: 1 Female: 6	n.s.
Mean height	158.7 ± 6.09 cm	152.4 ± 5.97 cm	n.s.
Systemic diseases	Hypertension: 3, Diabetes: 1 Dyslipidemia: 2, Angina pectoris: 1 Arrhythmia: 1, chronic kidney disease: 1	Hypertension: 3, Diabetes: 2 Angina pectoris: 1	n.s.
Walking ability before injury	Without walking aid: 10	Without walking aid: 5 With walking aid (a cane): 2	n.s.
Surgical methods	Femoral head replacement: 8 Open reduction and internal fixation: 2	Femoral head replacement: 2 Open reduction and internal fixation: 5	n.s.

n.s, indicates no significant difference between the indicated pairs.

* *P* < 0.05.

### 3.2 Effects of postoperative rehabilitation on each gait parameter

**Figure 2:**
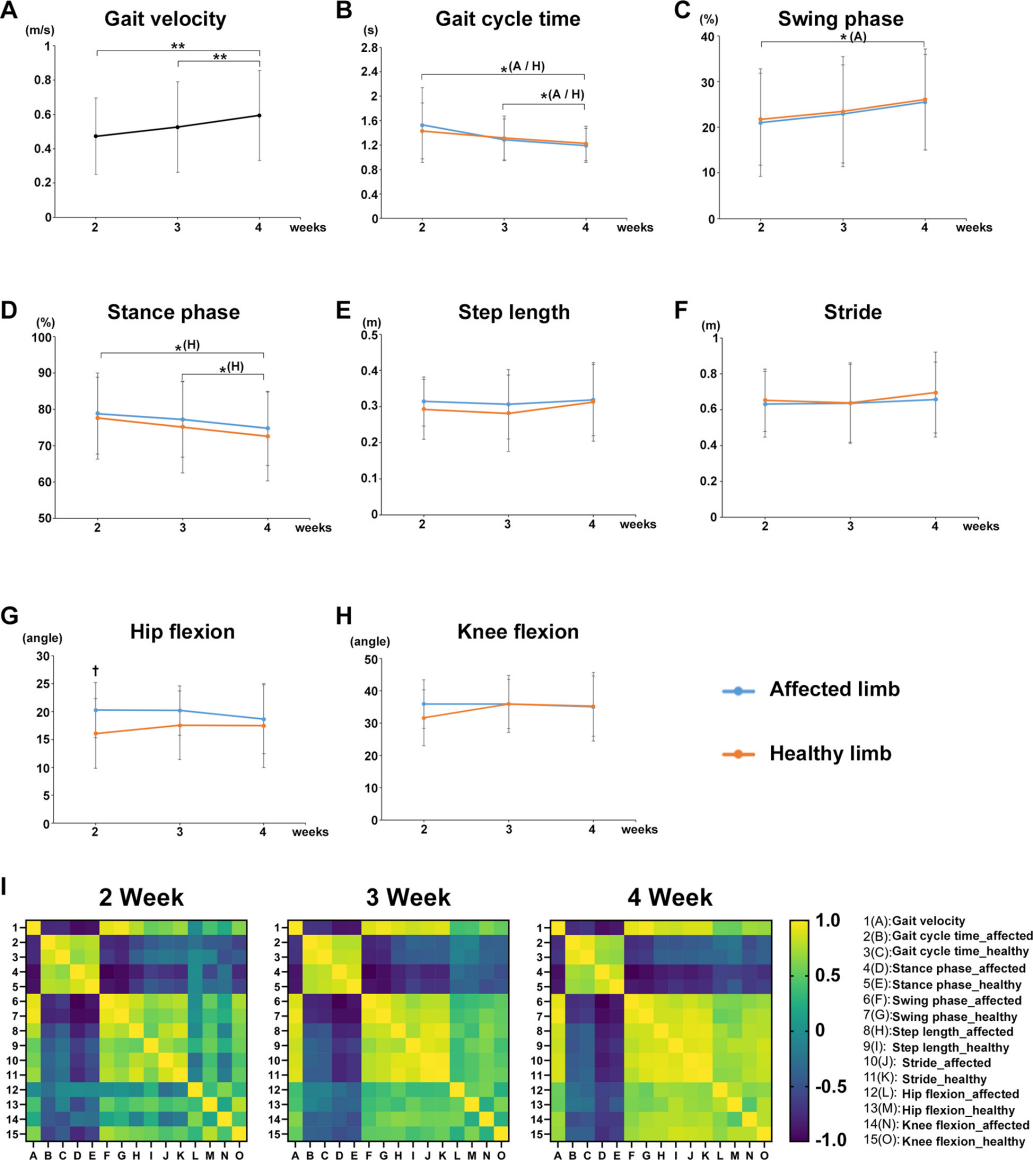
Effects of postoperative rehabilitation on each gait parameter. The mean value of each gait parameter for all participants from postoperative weeks 2–4. **(A)** gait velocity, **(B)** gait cycle time, **(C)** swing phase, **(D)** stance phase, **(E)** step length, **(F)** stride, **(G)** maximum hip joint flexion angle, and **(H)** maximum knee joint flexion angle. **(I)** Correlation heatmaps between the average values of gait velocity in all participants and the average values of each gait parameter from postoperative weeks 2–4. Black line, all participants; blue line, affected limb (operative side): orange line; healthy limb (non-operative side), A; affected limb, H; healthy limb, A/H; affected and healthy limbs, total participants, *n* = 17, mean ± SD, *; ANOVA with Shaffer *post-hoc* test for changes in gait parameters over time for all participants or affected and healthy limbs, †; Student's *t*-tests for gait parameters for all participants between affected and healthy limbs each week, *P *<* 0.05, **P *<* 0.01, and †*P* *<* 0.05.

The mean value of each gait parameter for all participants in both groups from postoperative weeks 2–4 was evaluated. Gait velocity significantly increased at weeks 3 (*P* = 0.0081) and 4 (*P* = 0.0081) compared with that at week 2 (Figure 2A). The gait cycle time of the affected and healthy limbs significantly decreased at week 4 compared with that at weeks 2 (affected limb, *P* = 0.0258; healthy limb, *P* = 0.0405) and 3 (affected limb, *P* = 0.0258; healthy limb, *P* = 0.0405) (Figure 2B). The swing phase of the affected limb significantly increased at week 4 compared with that at week 2 (*P* = 0.0355), and that for the healthy limb also tended to increase over time (Figure 2C). The stance phase of the affected limb tended to decrease over time, and that of the healthy limb significantly decreased at week 4 compared with weeks 2 (*P* = 0.0247) and 3 (*P* = 0.0247) (Figure 2D). No significant differences in the step length and stride of the affected and healthy limbs were found during the rehabilitation (Figures 2E,F). A significant difference in maximum hip joint flexion angle was observed between the affected and healthy limbs in the early postoperative period (week 2, *P* = 0.016, *r* = 0.5601) (Figure 2G). No significant differences were observed in the maximum knee joint flexion angle during the rehabilitation (Figure 2H). In addition, we created and verified correlation heatmaps over time between the average values of gait velocity in all participants and the average values of each gait parameter. As a result, the correlation coefficient between gait velocity and step length or stride or the maximum hip/knee flexion angles (none of these four gait parameters showed significant changes over time) showed a tendency to gradually show a positive correlation over time (Figure 2I).

### 3.3 Decrease in TUG over time and correlation with each gait parameter

**Figure 3:**
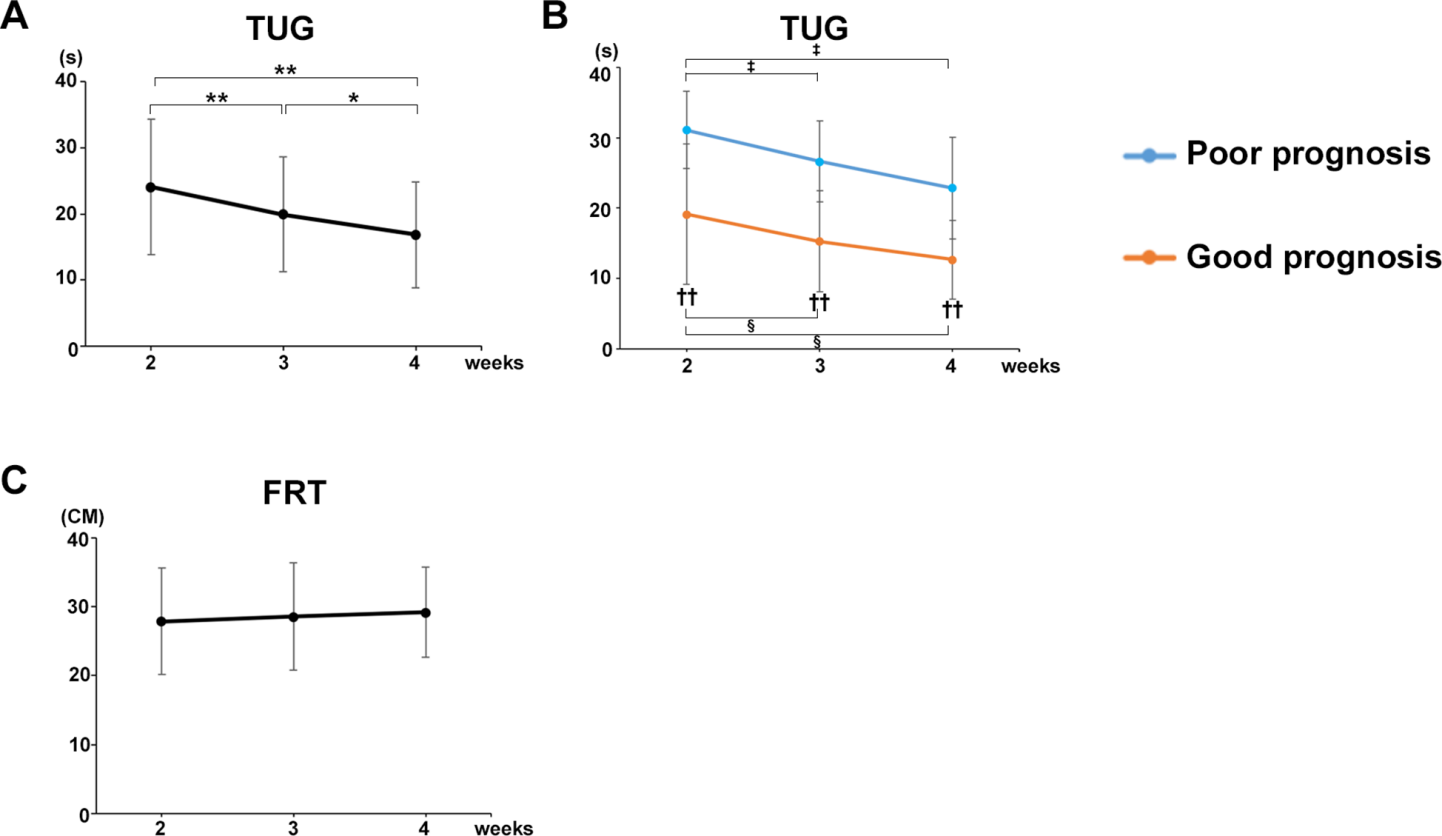
Decrease in TUG over time and correlation with each gait parameter. **(A)** Mean TUG values of all participants. **(B)** Mean TUG values of the good and poor gait prognosis groups. **(C)** Mean FRT values of all participants. Black line, all participants; blue line, poor gait prognosis group; orange line, good gait prognosis group, (total participants, *n* = 17; poor prognosis, *n* = 7; good prognosis, *n* = 10), mean ± SD, *; ANOVA with Shaffer *post-hoc* test for changes in TUG over time for all participants, ‡; ANOVA with Shaffer *post-hoc* test for changes in TUG over time for poor prognosis group, §; ANOVA with Shaffer *post-hoc* test for changes in TUG over time for good prognosis group, †; Student's *t*-tests for TUG between good and poor prognosis groups each week, *P *<* 0.05, **P *<* 0.01, ‡*P* *<* 0.05, §*P* *<* 0.05, and ††*P* *<* 0.01.

The mean TUG values of all participants in both groups significantly decreased at postoperative weeks 3 (*P* = 0.0004) and 4 (*P* = 0.0004) compared with that at week 2 (Figure 3A). TUG was significantly decreased at postoperative week 4 compared with that in both good gait prognosis (*P* = 0.0264) and poor gait prognosis (*P* = 0.0206) groups (Figure 3B). A significant difference in the TUG was observed between the two groups at postoperative weeks 2 (*P* = 0.00651, *r* = 05,959), 3 (*P* = 0.00267, *r* = 0.6665), and 4 (*P* = 0.00977, *r* = 0.6458) after surgery (Figure 3B).

Furthermore, the TUG was strongly correlated with the gait parameters in mean values of all participants at postoperative week 4 as follows (Table 3): Gait velocity (*P* = 0.0002, cor −0.782); step length (healthy limb, *P* = 0.0072, cor −0.625; affected limb, *P* = 0.0004, cor −0.757); stride (healthy limb: *P* = 0.0003, cor −0.771; affected limb: *P* < 0.0001, cor −0.824); swing phase (healthy limb, *P* = 0.0001, cor −0.808; affected limb, *P* = 0.0005, cor −0.753); stance phase (healthy limb, *P* = 0.0001, cor 0.805; affected limb, *P* = 0.0006, cor 0.747).

**Table 3:** The correlation between each gait parameter in mean values of all participants and TUG or OHS at postoperative week 4.

Gait parameters		TUG		OHS	
		*P*-value	cor	*P*-value	cor
Gait velocity		0.0002*	−0.782	0.0013*	0.714
Step length	healthy limb	0.0072*	−0.625	0.0314*	0.522
	affected limb	0.0004*	−0.757	0.0063*	0.634
Stride	healthy limb	0.0003*	−0.771	0.0026*	0.681
	affected limb	<0.0001*	−0.824	0.0004*	0.761
Swing phase	healthy limb	0.0001*	−0.808	0.0007*	0.740
	affected limb	<0.0001*	−0.824	0.0028*	0.678
Stance phase	healthy limb	0.0001*	0.805	0.0011*	−0.719
	affected limb	0.0006*	0.747	0.0034*	−0.668

Cor, correlation coefficient.

* *P* < 0.05.

By contrast, the mean FRT values of all participants in both groups did not change from postoperative weeks 2–4 (Figure 3C).

### 3.4 Comparison of gait parameters between poor and good prognosis groups

**Figure 4:**
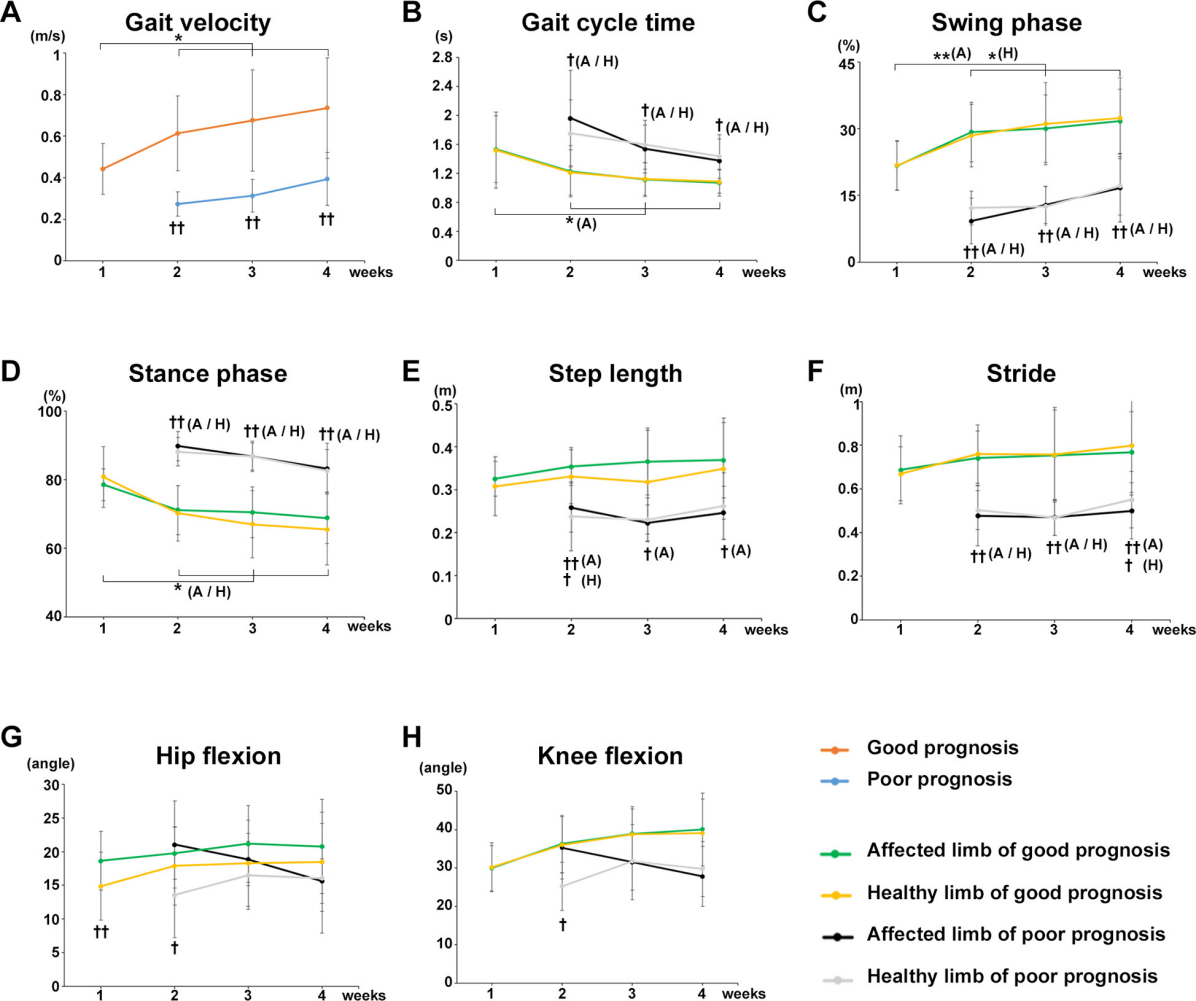
Comparison of gait parameters between poor and good prognosis groups. Comparison of the mean value of each gait parameter for the good and poor gait prognosis groups from postoperative week 1 to 4. **(A)** Gait velocity, **(B)** gait cycle time, **(C)** swing phase, **(D)** stance phase, **(E)** step length, **(F)** stride, **(G)** maximum hip joint flexion angle, and **(H)** maximum knee joint flexion angle. Orange line, good gait prognosis group; blue line, poor gait prognosis group; green line, affected limb of the good gait prognosis group; yellow line, healthy limb of the good gait prognosis group; blackline, affected limb of the poor gait prognosis group; gray line, healthy limb of the poor gait prognosis group, A; affected limb, H; healthy limb, A/H; affected and healthy limbs, (total participants, *n* = 17; poor prognosis, *n* = 7; good prognosis, *n* = 10), mean ± SD, *; ANOVA with Shaffer *post-hoc* test for changes in gait parameters over time for good and poor prognosis groups or affected and healthy limbs each good and poor prognosis groups, †; Student's *t*-tests for gait parameters between good and poor prognosis groups each week or affected and healthy limbs each good and poor prognosis groups each week *P < 0.05, **P < 0.01, †*P* < 0.05, and ††*P* < 0.01.

Participants who were unable to walk with a walker 1 week after surgery were allocated to the poor gait prognosis group and compared with a good prognosis group in terms of changes in each gait parameter. Gait velocity was significantly increased at postoperative weeks 2 (*P* = 0.0157), 3 (*P* = 0.0189), and 4 (*P* = 0.0157) compared with that at postoperative week 1 in the good prognosis group, whereas no significant change was observed in the poor prognosis group over time (Figure 4A). Significant differences in gait velocity were found between the poor prognosis and good prognosis groups at postoperative weeks 2 (*P* = 0.00014, *r* = 0.8529), 3 (*P* = 0.00099, *r* = 0.6968), and 4 (*P* = 0.00197, *r* = 0.6599) (Figure 4A).

The gait cycle time of the affected limb significantly decreased at postoperative weeks 2 (*P* = 0.0235), 3 (*P* = 0.0368), and 4 (*P* = 0.0277) compared with the postoperative week 1 in the good prognosis group, whereas no significant change was observed in the poor prognosis group over time (Figure 4B). A significant difference in gait cycle time was found between the poor and good prognosis groups at postoperative weeks 2 (affected limb, *P* = 0.02591, *r* = 0.612; healthy limb, *P* = 0.02354, *r* = 0.5959), 3 (affected limb, *P* = 0.01549, *r* = 0.6254; healthy limb, *P* = 0.00914, *r* = 0.6689), and 4 (affected limb, *P* = 0.0414, *r* = 0.5551; healthy limb, *P* = 0.02186, *r* = 0.6279) (Figure 4B).

The swing phase of affected and healthy limbs significantly increased postoperative weeks 2 (affected limb, *P* = 0.0080; healthy limb, *P* = 0.0177), 3 (affected limb, *P* = 0.0260; healthy limb, *P* = 0.0177), and 4 (affected limb, *P* = 0.02591; healthy limb, *P* = 0.0177) compared with postoperative week 1 in the good prognosis group, whereas no significant change was observed in the poor prognosis group (Figure 4C). A significant difference in the swing phase was noted between the poor and good prognosis groups at postoperative weeks 2 (affected limb, *P* = 0.00001, *r* = 0.8595; healthy limb, *P* = 0.00002, *r* = 0.8524), 3 (affected limb, *P* = 0.00003, *r* = 0.8151; healthy limb, *P* = 0.00009, *r* = 0.7824), and 4 (affected limb, *P* = 0.00133, *r* = 0.7298; healthy limb, *P* = 0.0013, *r* = 0.6914) (Figure 4C). On the other hand, the stance phase of affected and healthy limbs were significantly decreased at postoperative weeks 2 (affected limb, *P* = 0.0383; healthy limb, *P* = 0.0474), 3 (affected limb, *P* = 0.0462; healthy limb, *P* = 0.0206), and 4 (affected limb, *P* = 0.0383; healthy limb, *P* = 0.0206) in the good prognosis group, whereas no significant change was observed in the poor prognosis group (Figure 4D). A significant difference in the stance phase was found between the poor and good prognosis groups at postoperative weeks 2 (affected limb, *P* = 0.00001, *r* = 0.8398; healthy limb, *P* = 0.00004, *r* = 0.8472), 3 (affected limb, *P* = 0.00004, *r* = 0.8032; healthy limb, *P* = 0.00008, *r* = 0.847), and 4 (affected limb, *P* = 0.00178, *r* = 0.7096; healthy limb, *P* = 0.0008, *r* = 0.7032) (Figure 4D).

A significant difference in the step length was observed between the poor and good prognosis groups, respectively, postoperative weeks 2 (affected limb, *P* = 0.00344, *r* = 0.7111; healthy limb, *P* = 0.02678, *r* = 0.569), 3 (affected limb, *P* = 0.00023, *r* = 0.7511), and 4 (affected limb, *P* = 0.00425, *r* = 0.6325) (Figure 4E). A significant difference in stride was found between the poor and good prognosis groups at postoperative weeks 2 (affected limb, *P* = 0.00155, *r* = 0.7291; healthy limb, *P* = 0.00025, *r* = 0.7532), 3 (affected limb, *P* = 0.00188, *r* = 0.7403; healthy limb, *P* = 0.00224, *r* = 0.7388), and 4 (affected limb: *P* = 0.0029, *r* = 0.6514; healthy limb: *P* = 0.01258, *r* = 0.5554) after surgery (Figure 4F).

Regarding the joints' angles, no differences in maximum hip flexion angle and maximum knee joint flexion angle were observed between the poor and good prognosis groups (Figures 4G,H). However, in both groups, significant differences in the maximum hip joint flexion angles were observed between the affected and healthy limbs in the early postoperative period (good prognosis group week 1, *P* = 0.00169, *r* = 0.827; poor prognosis group week 2, *P* = 0.020, *r* = 0.7875), and the difference gradually decreased (Figure 4G). Furthermore, in the poor prognosis group, a significant difference in the maximum knee joint flexion angle was observed between the affected and healthy limbs in the early postoperative period (week 2, *P* = 0.034, *r* = 0.7439) (Figure 4H).

### 3.5 Increase in OHS over time and correlation with each gait parameter

**Figure 5:**
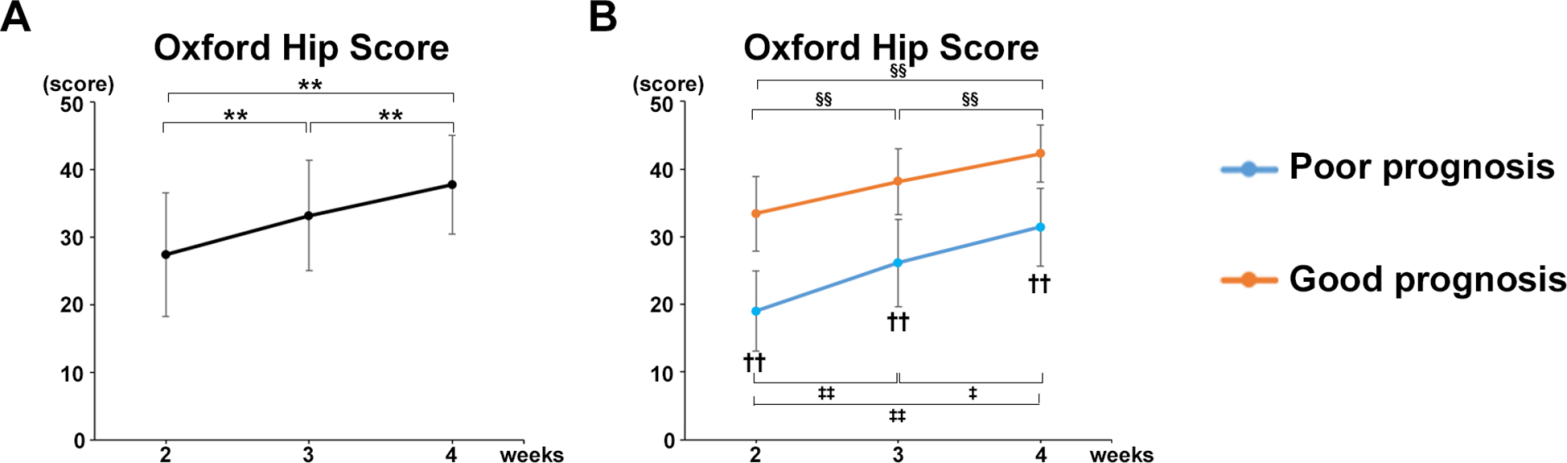
Increase in OHS over time and correlation with each gait parameter. **(A)** Mean OHS values of all participants. **(B)** Mean OHS values of the good and poor gait prognosis groups. Black line, all participants; blue line, poor gait prognosis group; orange line, good gait prognosis group, (total participants, *n* = 17; poor prognosis, *n* = 7; good prognosis, *n* = 10), mean ± SD, *; ANOVA with Shaffer *post-hoc* test for changes in OHS over time for all participants, ‡; ANOVA with Shaffer *post-hoc* test for changes in OHS over time for poor prognosis group, §; ANOVA with Shaffer *post-hoc* test for changes in OHS over time for good prognosis group, †; Student's *t*-tests for OHS between good and poor prognosis groups each week, *P *<* 0.05, **P *<* 0.01, ‡*P* *<* 0.05, ‡‡*P* *<* 0.01, §§*P* *<* 0.01, and ††*P* *<* 0.01.

The OHS in all participants in both groups increased significantly at postoperative week 3 and 4 compared with that at weeks 2 (*P* < 0.0001) (Figure 5A). The OHS significantly increased at postoperative week 4 compared with weeks 2 (*P* = 0.0003) and 3 (*P* = 0.0003) in the good prognosis group. The OHS also significantly increased at week 4 compared with weeks 2 (*P* = 0.0043) and 3 (*P* = 0.0116) in the poor prognosis group (Figure 5B). A significant difference in OHS was observed between the poor and good prognosis groups at postoperative weeks 2 (*P* = 0.00024, *r* = 0.7976), 3 (*P* = 0.0016, *r* = 0.7518), and 4 (*P* = 0.0015, *r* = 0.7582) (Figure 5B).

Furthermore, the OHS was strongly correlated with the gait parameters in mean values of all participants at postoperative week 4 as follows (Table 3): Gait velocity (*P* = 0.0013, cor 0.714); Step length (healthy limb, *P* = 0.0314, cor 0.522; affected limb, *P* = 0.0063, cor 0.634); Stride (healthy limb, *P* = 0.0026, cor 0.681; affected limb, *P* = 0.0004, cor 0.761); Swing phase (healthy limb, *P* = 0.0007, cor 0.740; affected limb, *P* = 0.0028, cor 0.678); Stance phase (healthy limb, *P* = 0.0011, cor −0.719; affected limb, *P* = 0.0034, cor −0.668).

### 3.6 Effect of rehabilitation gait training intervention for the same period on gait parameters between poor and good prognosis groups

**Figure 6:**
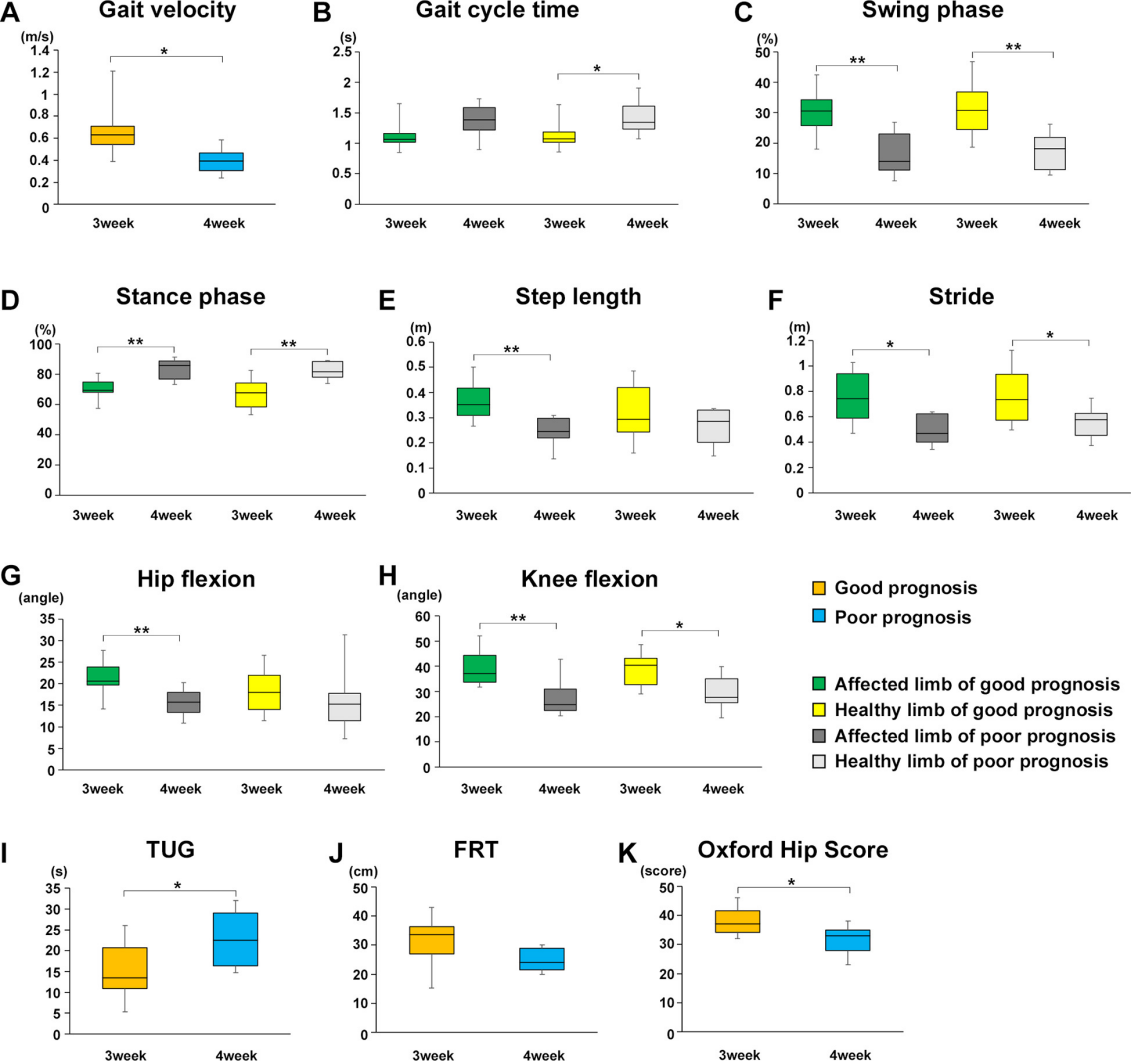
Effect of rehabilitation gait training intervention for the same period on gait parameters, TUG and OHS between poor and good prognosis groups. Comparison of the mean value of each gait parameter for the good and poor gait prognosis groups 3 weeks after starting walking training (good prognosis group; 3 weeks after surgery, poor prognosis group; 4 weeks after surgery). **(A)** Gait velocity, **(B)** gait cycle time, **(C)** swing phase, **(D)** stance phase, **(E)** step length, **(F)** stride, **(G)** maximum hip joint flexion angle, **(H)** maximum knee joint flexion angle, **(I)** TUG, **(J)** FRT, and **(K)** OHS. Orange line, good gait prognosis group; blue line, poor gait prognosis group; green line, affected limb of the good gait prognosis group; yellow line, healthy limb of the good gait prognosis group; blackline, affected limb of the poor gait prognosis group; gray line, healthy limb of the poor gait prognosis group (total participants, *n* = 17; poor prognosis, *n* = 7; good prognosis, *n* = 10), mean ± SD, *; Student's *t*-tests for gait parameters, TUG and OHS between good and poor prognosis groups or affected and healthy limbs each good and poor prognosis groups, *P < 0.05, **P < 0.01.

On the other hand, since we divided the participants into two groups based on whether they could independently walk at week 1 (poor and good), the total walking training periods of the two groups were different. In the poor prognosis group, participants who were unable to walk at week 1 were evaluated at week 4, which means that they only had 3 weeks of walking rehabilitation. To address this point, we compared the two groups again at 3 weeks postoperatively for the good prognosis group and 4 weeks postoperatively for the poor prognosis group, with the same 3-week postoperative rehabilitation period for both groups. As a result, a significant difference in the gait parameters was observed between the poor and good prognosis groups as follows (Figure 6): Gait velocity (*P* = 0.0138, *r* = 0.5842); Gati cycle time (healthy limb, *P* = 0.0279, *r* = 0.5321); Swing phase (healthy limb, *P* = 0.0038, *r* = 0.6621; affected limb, *P* = 0.0028, *r* = 0.6778); Stance phase (healthy limb, *P* = 0.0023, *r* = 0.6877; affected limb, *P* = 0.0033, *r* = 0.6699); Step length (affected limb, *P* = 0.0043, *r* = 0.6551); Stride (healthy limb, *P* = 0.0405, *r* = 0.5011; affected limb, *P* = 0.0115, *r* = 0.5963); Hip flexion angle (affected limb, *P* = 0.0077, *r* = 0.6214); Knee flexion angle (healthy limb, *P* = 0.0173, *r* = 0.5685; affected limb, *P* = 0.0076, *r* = 0.6226). Similarly, a significant difference in the TUG time and OHS, but not FRT were observed between two groups as follows (Figures 6I–K): TUG (*P* = 0.0496, *r* = 0.4828); OHS (*P* = 0.0192, *r* = 0.5609).

## 4 Discussion

In this study, gait was analyzed using Kinect over time during acute rehabilitation after PFF surgery, and objective gait evaluation and gait prognosis prediction factors were examined by comparing the poor and good prognosis groups. Increased gait velocity, decreased gait cycle time, increased swing phase, decreased stance phase, increased maximum hip joint flexion angle were observed during the postoperative rehabilitation. Rehabilitation also contributed decreased TUG, dynamic balance indicator, and increased OHS score, hip joint pain/ADL index, in all participants over time. The poor gait prognosis group who was unable to walk with a walker at postoperative week 1 showed worse gait parameters, gait velocity, swing/stance phases, step length, stride, mean TUG and OHS value compared with the good prognosis group over time. In particular, a significant difference was observed in the swing/stance phases of the affected or healthy limbs between the good and poor prognosis groups. Furthermore, decreased TUG and increased OHS strongly correlated with gait velocity, step length, stride, and swing/stance phases at postoperative week 4. Therefore, to achieve a good walking outcome, walking training should be initiated within 1 week after surgery to increase the weight-bearing rate of the affected limb. To the best of our knowledge, this is the first report of the objective gait analysis study based on posture estimation using Kinect for the acute phase of postoperative PFF.

Reduction of acute pain in patients with PFF is important for early acquisition of walking ability for patients who were able to walk independently before the injury (17). In this study, the significant increase of postoperative OHS in all participants in both groups who could walk before surgery, correlated with the changes in each gait parameter, gait velocity, step length, stride, and swing/stance phases. Furthermore, compared with the good prognosis group over time, those in the poor gait prognosis group who were unable to walk with a walker at postoperative week 1 had worse postoperative OHS as well as each gait parameter. These results indicated that appropriate hip joint pain control after the acute phase of PFF surgery, particularly during the mobilization period at postoperative week 1, is important for gait prognosis. Interestingly, although there was no significant difference for surgical methods in both groups, participants who underwent internal fixation tended to be classified into the poor prognosis group and were more likely to present chronic postoperative pain (Table 2). It has been reported that patients with PFF underwent internal fixation are more likely to experience decrease load-bearing rates with the affected limb on the acute rehabilitation training period because of persistent surgical site pain than those who underwent femoral head replacement (18). Therefore, patients who underwent internal fixation require rehabilitation under more intensive pain management.

Predictors affecting walking ability in the acute phase after PFF surgery include age, degree of dementia, and pre-injury walking ability (19). We focused on changes in gait post-surgery, minimizing medical history factors that could influence outcomes. Despite efforts to exclude confounding factors, our data showed that younger age participants were significantly more likely to be in the group with good prognosis (Table 2). Although this study was a statistical analysis of two small sample size groups with overlapping age distributions, age might be a key predictor that affect walking prognosis.

Clinical evaluations affecting gait prognosis during postoperative rehabilitation include early weight-bearing and gait training initiation (20). Early weight-bearing reportedly reduces mortality rates after hip fracture surgery (21). In our study, swing and stance phases differed significantly between poor and good prognosis groups and the increased swing phase and decreased stance phase correlated strongly with OHS scores. In the poor prognosis group, no significant changes in the swing/stance phase were observed over time. The swing phase of the healthy limb indicates single limb support on the affected limb. Effective hip pain control and increased weight-bearing are crucial for positive outcomes.

In the poor prognosis group, insufficient time for gait training impacted weight-bearing and load balance. The maximum flexion angle of the hip/knee in early postoperative periods was higher on the affected limb, likely due to compensatory mechanisms (Figures 4G,H). After surgery for PFF, hip and knee extension on the affected side is restricted, and the patient's leg tends to be in a flexed position (22). Restriction of hip/knee extension leads to flexed limb positions, exacerbated by prolonged bedridden periods. Early extension exercises are necessary. Compared to the good prognosis group, the poor prognosis group showed longer bedridden periods and extended restriction of hip/knee extension. Compensatory gait was observed at two weeks, with differences in flexion angles resolving within 3–4 weeks as training progressed. Postoperative hip extension restriction affected gait parameters like velocity and stride, resulting in poorer outcomes. Our results suggest that early gait training within one-week post-surgery, focusing on hip pain control and weight-bearing, can improve rehabilitation outcomes and facilitate early home return.

In this study, TUG and FRT were measured as dynamic balance indicators. The mean TUG values of all participants in both groups significantly decreased over time at the early phase of the rehabilitation training period after surgery. In particular, the good gait prognosis group had a higher mean TUG than the poor prognosis group, which correlated with each gait parameter; four weeks after surgery (the time point of discharge from hospital), the mean TUG was 13.18 ± 7.91 in the good gait prognosis group, and 22.91 ± 5.70 s in the poor prognosis group. Patients who have a TUG of ≥24 s at the time of discharge after PFF surgery have a higher rate of recurrent falls within 6 months (23). In this study, the mean TUG value in the good gait prognosis group at the time point of discharge was lower than the cutoff value. Thus, postoperative gait training by focusing on the correlated gait parameters (gait velocity, step length, stride, and swing/stance phase of the affected and healthy limbs) may prevent recurrent falls. By contrast, the FRT did not change over time. The average FRT of all participants in both groups maintained above the elderly people's fall cutoff value [<15.3 cm (24)], on the mean value from postoperative weeks 2–4 (Figure 3C). Generally, FRT reflects the ability to move the center of gravity of the body by the standing position within the same base of support, and this is different from walking motion, which evaluates the postural control function of the center of gravity movement accompanied by changes in the base of support (25). Because all study participants had adequate walking ability before injury, it is likely that strength in the triceps surae and erector spine muscles, which mainly affect FRT, was maintained postoperatively.

Finaly, we set the rehabilitation intervention periods for both groups to 3 weeks, and found that significant differences were observed in each gait parameter, TUG, and OHS between the good and poor gait prognosis groups, despite the same rehabilitation training period. Therefore, it seems likely that early initiation of gait training affects postoperative walking prognosis, regardless of the duration of postoperative independent walking training.

We have previously reported that the gold-standard system of motion capture and Kinect can perform equivalent gait analysis in healthy subjects (11). In the acute phase after proximal femoral fracture surgery, marker placement of the gold standard system seems difficult due to postoperative pain. Therefore, Kinect, a non-invasive, easily prepared, markerless gait analysis device, can be easily used in many facilities.

## 5 Limitations

This study had some limitations. First, this study was a single-center study with a small sample size. Although the sample size was small, parameters that showed significant differences in the between-group comparisons had large effect sizes in power analysis. Second, because we wanted to examine gait changes purely due to the influence of the PFF, we employed strict exclusion criteria. Third, in this study, we used the *Y*-axis (vertical) and the hip–knee vector on the *Y–Z* plane to calculate the maximum hip flexion angle based on our previous study (11). On the other hand, the 3D model calculated by the RGB depth camera based on the distance to each joint point can extract good linear and angular parameters. Although we did not use it in this study, the hip flexion angle could be calculated more accurately if it was calculated from the spine axis vector and hip-knee joint vector. In the acute postoperative phase of PFF, the patient's trunk tends to lean forward when trying to support the upper limbs while walking with a cane or walker. In this study, when a walker with strong upper limb support was used approximately 1 to 2 weeks after surgery, the calculated maximum hip flexion angle may have been lower than the actual maximum hip flexion angle. Fourth, Microsoft Kinect v2® system was out of production in 2017. Orbbec Femto Bolt® is now available as a successor to Kinect v2®. Compared to Kinect v2®, Orbbec Femto Bolt® possess better RGB depth data alignment accuracy and enables to generate more accurate 3D skeletal models (https://www.orbbec.com/documentation/comparison-with-azure-kinect-dk/). Finally, patient-reported outcomes specific to quality of life were not investigated.

## 6 Conclusions

Objective gait analysis using Kinect in the acute phase of postoperative rehabilitation after PFF surgery revealed that appropriate hip joint pain control and early weight-bearing on the affected limb, which was able to walk using a walker 1 week after PFF surgery, are key factors for obtaining a good gait prognosis and preventing recurrent falls. We believe that rehabilitation interventions targeting these key factors will lead to an extension of healthy life expectancy in the elderly.

## Data Availability

The raw data supporting the conclusions of this article will be made available by the authors, without undue reservation.
